# Theory of Cooperative-Competitive Intelligence: Principles, Research Directions, and Applications

**DOI:** 10.3389/fpsyg.2020.02220

**Published:** 2020-09-11

**Authors:** Robert Hristovski, Natàlia Balagué

**Affiliations:** ^1^ Complex Systems in Sport Research Group, Faculty of Physical Education, Sport and Health, Ss. Cyril and Methodius University, Skopje, Macedonia; ^2^Complex Systems in Sport Research Group, Institut Nacional d’Educació Física de Catalunya (INEFC), Universitat de Barcelona (UB), Barcelona, Spain

**Keywords:** intelligence, cooperative-competitive intelligence, sport intelligence, game intelligence, embodied intelligence, affordances, perception-action, entropy

## Abstract

We present a theory of cooperative-competitive intelligence (CCI), its measures, research program, and applications that stem from it. Within the framework of this theory, satisficing sub-optimal behavior is any behavior that does not promote a decrease in the prospective control of the functional action diversity/unpredictability (D/U) potential of the agent or team. This potential is defined as the entropy measure in multiple, context-dependent dimensions. We define the satisficing interval of behaviors as CCI. In order to manifest itself at individual or team level, this capacity harnesses properties such as degeneracy, pleiotropy (pluri-potentiality), synergies, and metastability. Intelligence is embodied because intelligent behavior is deeply dependent on body functionalities, defined as entropy measures. We base our theory on three principles: (a) relativity of functional entropy/information in agent (team)-environment systems, (b) tendency toward the satisficing level of D/U potential, and (c) tendency toward the non-decreasing D/U potential. The conjunction of these three principles provides existence of sub-optimal behaviors associated with CCI. First, we deal with the problem of how to reduce multidimensional behavior to a concept that accounts for the vast set of scenarios in which CCI is manifested. Secondly, we define and discuss the three interacting principles that underpin CCI behavior as well as providing an outline for a future CCI research program supported by agent-based modeling and empirical research. Finally, we provide some preliminary practical issues that stem from the theory.

## Introduction

Concepts such as game intelligence, sport intelligence, or technical/tactical intelligence are common in the scientific literature and in discussions among practitioners but are used vaguely and have rarely been elaborated (e.g., Gould et al., 2002; Blue, 2009; Memmert et al., 2010; Rosslee, 2014; Lennartsson et al., 2015). These concepts have been used primarily in team sports, while their use for individual sports is mostly absent altogether (with the exception of Blue, 2009 for golf-specific intelligence). Sport intelligence has been predominantly considered from the traditional trait theory perspective and from explanatory patterns belonging to and used eminently by the information-processing theory. For example, Gould et al. (2002) defined sport intelligence as a highly developed set of traits, such as decision-making, innovativeness, and quick learning. Memmert et al. (2010) used the traditional, sport-contextualized definition of intelligence as convergent tactical thinking, or more precisely, as the ability to find the best solution (e.g., best positioning). Recently, a thoroughly developed “psychometric trait” model of sport intelligence was suggested (Rosslee, 2014). This model construes sport intelligence as a set of six interacting sub-systems spanning over different levels, starting from the neuro-physiological up to the metaphysical level. On the other hand, psychometric research on collective intelligence traits (e.g., Woolley et al., 2010) have revealed that individual intelligence contributes far less than the properties of interpersonal interactions. This means that a psychometric (or indeed, any other lower level, such as neuro-physiological or genetic) approach to intelligence reveals a high level of dependence in the understanding of intelligence. Hence, a general conceptual framework of intelligence, valid for both the individual and the collective level, seems hard to obtain within these kinds of approaches. This state of affairs clearly points to a need for a more general conceptualization of intelligence in cooperative-competitive environments, which would be valid for the individual as well as the collective level of action. In other words, level-dependent variables, although important for each level, have to be treated as specific rather than general determinants of CCI.

Another aspect of game intelligence was captured by constructing a theoretical game model (Lennartsson et al., 2015). Within the framework of this approach, the fundamental idea is the concept of *potential*, that is, the difference between the probability of the offense scoring the next goal and the probability that the next goal is scored by the defense. Authors have obtained optimal strategies for both offense and defense, and one main result is that the optimal defensive strategy exists when the maximum potential of all offensive strategies is minimized.

Based on the above, it becomes clear that intelligence in competitive-cooperative environments, such as sport, can be conceptualized differently depending on the level at which it is defined (personal or collective), or on some limited behavioral properties that lack generality. This is the main reason why we have adopted a more general approach to intelligence in this paper as the tendency of living systems and their dynamic social structures (e.g., teams) to *evade* and *escape* states of reduced possibilities in what we call functional action diversity/uncertainty (D/U) potential, where the potential is expressed through the entropy concept (Hristovski, 2017). Under D/U potential, we understand action diversity/uncertainty, which consists not only of richness of the functional coordinative patterns (i.e., functional classes of action or movement forms) of the agent and/or among agents but also of the diversity/uncertainty potential in timing resolution, speed, and other skill parameters. For example, larger entropy in the variable of accuracy or acuity means larger resolution of perception-action. A constant space with a finer-grained structure, or higher sensitivity to details, has larger entropy because of greater discriminatory ability (see Gibson and Gibson, 1955; Araújo et al., 2019). In a similar vein, D/U potential may be based on degeneracy, i.e., the capacity of agents and teams to attain a similar outcome by structurally different components, e.g., Edelman and Gally (2001), but can also be based on the capacity to functionally change the intended outcomes. Hence, these concepts are not necessarily reducible to degeneracy.

In the text that follows, we offer a general conceptualization of what we call cooperative-competitive intelligence (CCI) in order to capture current definitions of game intelligence as special cases of CCI. CCI would include a vast set of behaviors that exist in sports science literature under different names, such as: sport intelligence, game intelligence, and technical/tactical intelligence. However, it may also include a wider set of behaviors outside the immediate sports performance realm, such as strategic planning.

The CCI term is chosen because it does not tend to define intelligent behavior solely at the scale of sports performance (e.g., a competition or a match) but also captures the longer-term tendencies of strategic behavior in systems that contain cooperative-competitive interactions in general. Keeping in mind that the systems we plan to discuss are multidimensional complex adaptive systems, the first question that comes to mind is: how can we dimensionally reduce the multidimensional behavior to a concept that can be useful in a principled way in accounting for the vast set of scenarios in which CCI is manifested? Obviously, this problem needs a selection of what can be called a “common conceptual currency.”

## Entropy as a Common Conceptual Currency

In recent research, entropy as a partial measure of performance has been extensively used either in individual or in collective sports. Researchers have investigated a large set of variables in many sports disciplines (e.g., Hristovski et al., 2006; Passos et al., 2009; Fewell et al., 2012; Vilar et al., 2013; Couceiro et al., 2014; Silva et al., 2016; Castellano et al., 2017; Gonçalves et al., 2017; Neuman et al., 2018; Lopes and Tenreiro Machado, 2019, 2020), showing that behavioral entropy, as measured by different entropy measures, has considerable effect on a number of teams and individual performance indexes. These immensely important results seem to point to a possible unifying explanation of how entropy, as a property of these systems, enters and becomes relevant for performance. We concur with the claim that behavioral entropy is a highly relevant concept for sports performance. In the text that follows, we will explain the relevance of the entropy concept for performance by showing that it underpins the CCI concept *in a specific way*.

The main reason why CCI can be defined in entropy terms is because it sufficiently unifies different measures of variability (e.g., variance, range, etc.), that is, variability can be put under the same measure. Moreover, it is often expressed as a logarithm of a spatial construct, line, surface, volume, and any scale whether physical or formal (i.e., formal scales in psychology or physiology can be cast in spatial terms). In thermodynamics, the dependence of entropy on variability and space variables are given in one of the basic relations between entropy and temperature (i.e., variability) and volume (space) (Balesku, 1975). Hence, an entropy value can be ascribed to any construct that can be expressed as a variability and/or a space. In this way, entropy enables us to work with a “*common currency*” in different dimensions. For example, space creation and occupation, number of possible passes, and agility can be all expressed as entropy.

In the research on perception-action, it has been convincingly argued that the information picked up by agents to control their actions can be cast as a co-variance between the distal properties of environment and the structured energy array that further co-varies with the perceptual systems of agents (Segundo-Ortin et al., 2019). Because of the co-variance relation, the ecological information can be quantified as Shannon information (defined as a reduction of entropy; e.g., de Carvalho and Rolla, 2020).

In the text below, we define and discuss the three interacting principles that underpin CCI behavior. We first discuss the relativity of the information-entropy principle and show what the adaptation of agents and teams to their environment means in terms of the increment of the functional integrative information of the system. The integrative information of the system is seen by the external observer as behavioral D/U potential. The sufficing variability principle then sets a limit to the growth of the D/U potential and is manifested as a dynamic entity dominantly constrained by the richness of environmental perturbations. The tendency toward non-decreasing action D/U potential unifies the manifestations of CCI in different dimensions and some aspects of creative behavior. Finally, we discuss some aesthetic, practical consequences and outline a research program that stems from this conceptualization of CCI. To help the interested reader grasp some of the more abstract ideas, suitable examples are provided in each subheading.

## Principles of the Theory

### Principle 1: The Relativity of Information^1^ Entropy. Non-functional and Functional Action Diversity/Uncertainty Potential

The principle of D/U potential (Hristovski, 2017) captures two moments. First, it captures important aspects of the transition from non-functional to functional action, diversity/uncertainty of the system (agent or team). Second, it captures the relativity of the role of functional action diversity/uncertainty potential for the system when seen, on the one hand, from within, and on the other, by an external observer. The term *potential* signifies that the diversity or uncertainty of actions need not be manifested always and everywhere. When the context allows, the system may attain its goals using highly repetitive actions. The term “potential” means a space of individual or collective action properties, which, *when needed*, can be organized in order to attain a certain well-defined goal or chain of sub-goals. It also has a wider meaning than repertoire of movement forms, including perceptual and other psychomotor abilities.

This principle contends that the practice-induced transition from non-functional to functional D/U potential from the perspective of the agent or team (perspective from within) represents gaining integrative information and greater *within-system* certainty. Seen, however, from the external observers’ (e.g., opponent’s) perspective, it represents gaining functional entropy or functional uncertainty. This is because in a finite configuration space, the sum of the entropy and information is constant (Layzer, 1975). This means that before the occurrence of some event, its entropy (i.e., degree of uncertainty) is equal to the information one obtains after its occurrence. A gain in information is always compensated by loss in entropy and vice versa (Layzer, 1975; Serdyukov, 1987). Hence, according to this, the training process is conceptualized as a conversion of entropy into stable integrative information structured by different psychomotor dimensions (Hristovski, 1989). Integrative information is defined as information that arises from the couplings among goal-directed actions of the system. The behavior of agents in a deterministic and stable environment is then formulated as a variation principle of the least entropy (uncertainty) action. From this, it follows that in highly stable and repetitive (i.e., predictable) environments, adaptive systems will converge to a minimum uncertainty by minimizing the irrelevant action variations. However, even so-called “individual” sport competitions rarely offer highly stable environments. On the contrary, competitions create conditions where the highly demanding non-cooperative behavior of the environment is the rule rather than the exception. In such non-deterministically changing non-cooperative environments, agents (players and teams) must develop high D/U potential to increase their fitness and survival possibilities. This means that the adaptation process on long time scales, such as years, rests on a tendency of permanent increase in the D/U potential which affords the ultimate goal, the survival (winning) of the system in sports environments. In our view, therefore, cooperative intelligence would crucially depend on how the agent or team manages the adequate level of integrative information within its boundaries and the entropy (unanticipatedness or uncertainty) potential for the opponent, while being continually under their (environmental) perturbing influences. Between-team competitive intelligence would depend on the abilities of the agent or team to suppress the opponents’ integrative information and increase the non-functional entropy. Importantly, within-team competitive intelligence would be higher, if the intra-team competition brought about larger integrative information and larger entropic (uncertain) behavior potential for the opponents.

The principle is general, but let us cast it in a more familiar form for the reader, in terms of synergies and the process of reducing the bad and increasing the functional (good) variability (Latash, 2008). This example is especially important to make a distinction between non-functional and functional D/U potential. The former is present mostly in novices, and the latter, in experts. Synergy has been defined as the capacity of reciprocal compensatory intervention of component variables V_1_–V_n_ in order to maintain the achievement of certain goal or performance variables (Latash, 2008). The co-varying and reciprocally compensating components induce necessarily a dimension reduction of the system. It has mostly been exploited in motor control literature (Schöner, 1995; Scholz and Schöner, 1999; Latash, 2008; Maldonado et al., 2018) and to a lesser degree in interpersonal, social systems literature (Dodel et al., 2010; Riley et al., 2011; Passos et al., 2018).

**Figure 1:**
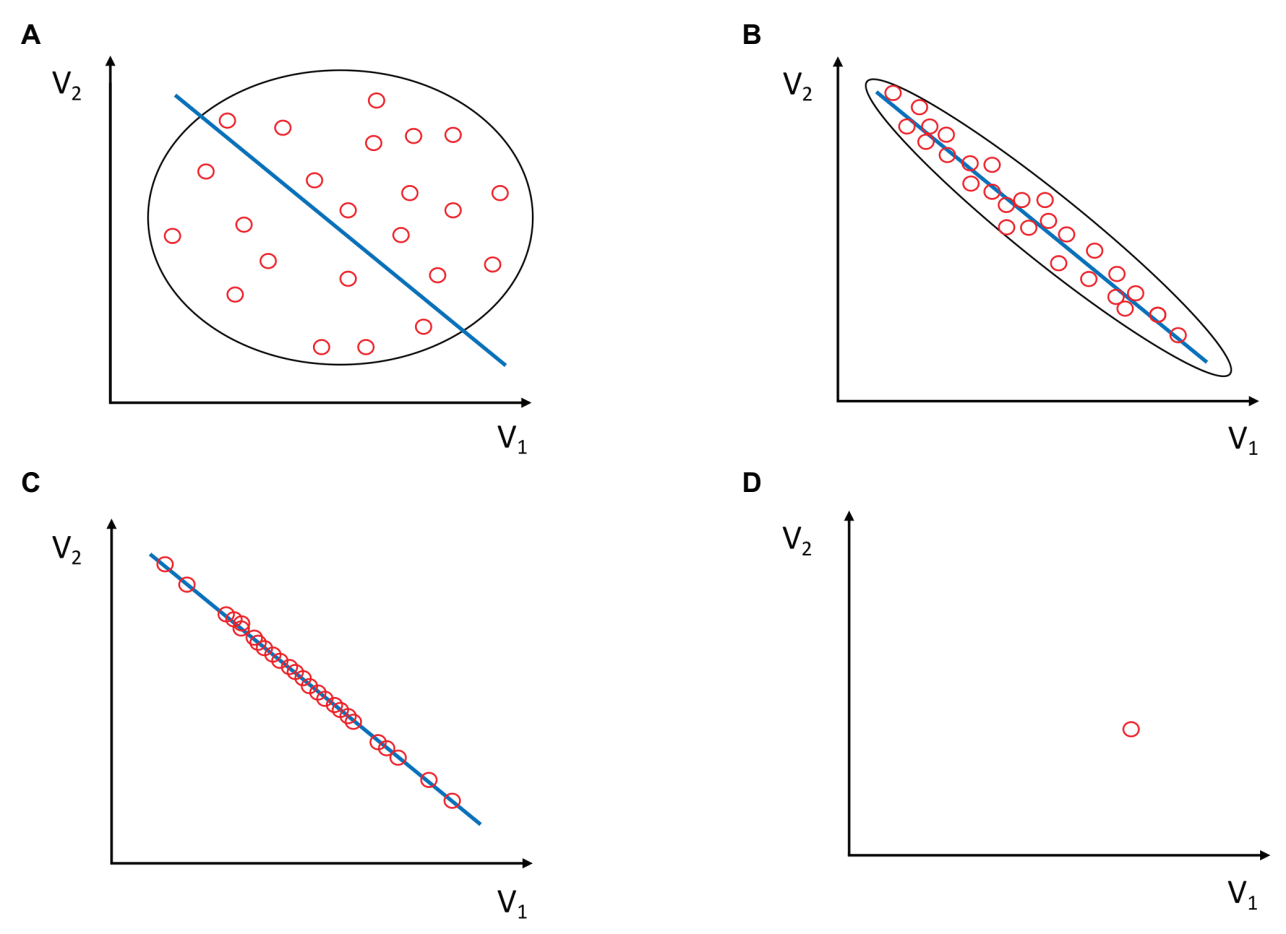
Task solutions (i.e., behaviors) are given as combinations of component variables V_1_ and V_2_ as red ovals. Task solutions that satisfy goal constraints are given as a blue line. These solutions are functional task solutions. Intuitively, entropy decreases and information increases with the higher condensation of red ovals along the blue line. This is captured by the shape of the oval on the scatterplot. **(A)** Component variables V_1_ and V_2_ do not efficiently co-vary. They seldom lie along the blue line. Consequently, task solutions rarely satisfy goal constraints. Entropy H is maximal, and hence, common integrative information within the system is minimal. The variability and uncertainty of individual or team behavior are also maximal, but rarely functional (i.e., positioned at the blue line). Thus, the diversity/uncertainty (D/U) potential is small. **(B)** Task solutions fit well with the blue line, which consists of the values of combinations of V_1_ and V_2_, which satisfy the goal constraints. There is high, although not complete, functional co-variation of component variables V_1_ and V_2_. The variability along the blue line is larger than the variability perpendicular to it and, therefore, the functional D/U potential is larger than that in panel A. There is lower than maximal entropy in the system and, hence, common integrative information within the system is present. **(C)** All the variability of task solutions lies on the blue line, meaning they all satisfy the goal constraints. Component variables maximally and functionally co-vary. This is the case of maximal functional D/U potential. **(D)** Only one combination of component variables V_1_ and V_2_ exists and satisfies the goal constraint. All trials are accumulated in the same oval. Neither variability nor functional diversity exists in this case, meaning that D/U potential is zero. This case corresponds to a maximally stable action in a deterministic environment, e.g., automatic robot devices in car factories. The entropy of the system (H) is zero and the integrative information (I) is maximal.

In the light of the principle of entropy-information relativity, agent or team adaptation may be conceptualized as an increasing disagreement between the external observer and the agent (or team) performing a task on the level of the *functional* uncertainty of the agent’s or team’s future policy. For simplicity, let us assume that a certain policy has to satisfy a well-defined stable task goal constraint^2^. As depicted in Figures 1A–C, the process of adaptation may be portrayed as a sequence A->B->C; that is, as an ongoing condensation of configurations of the component actions given by variables V_1_ and V_2_ on the manifold (the blue line) signifying the increased frequency of attaining the task goal. One can consider that each red oval represents a task realization (a trial), which was achieved by some configuration of component variables V_1_ and V_2_. Panel A would represent a case where the agent, dyad, or the team very rarely comes close to attaining the goal. Accordingly, panel C would, therefore, represent an ideal case in which all trials of the agent, dyad, or the team attain the goal (e.g., scoring a point or making a successful pass).

In Figure 1, the functional variability spreads along the blue line and the non-functional variability spreads in a direction perpendicular to the blue line. One may understand it as a goodness of fit between the configurations of component values V_1_ and V_2_ and the blue line which represents the subset of configurations of V_1_ and V_2_ which satisfy the goal constraints. In other words, it represents how good the co-varying and reciprocally compensating combinations of components fit the goal. If V_1_ and V_2_ lie anywhere along the blue line, their synergy satisfies the goal constraint. If they lie far from the blue line, there is no functional synergy. The goal is far from being attained. Hence, there may be a large number of combinations of component actions that satisfy the goal constraints, not only one. In multidimensional spaces, more than two independent component variables may also create synergies (Latash et al., 2007). The component variables V_1_–V_n_ may be intrapersonal (e.g., muscle activations, joint angles, moments of inertia, etc.) or interpersonal variables (see Black et al., 2007; Dodel et al., 2010; Riley et al., 2011; Passos et al., 2018).

**Figure 2:**
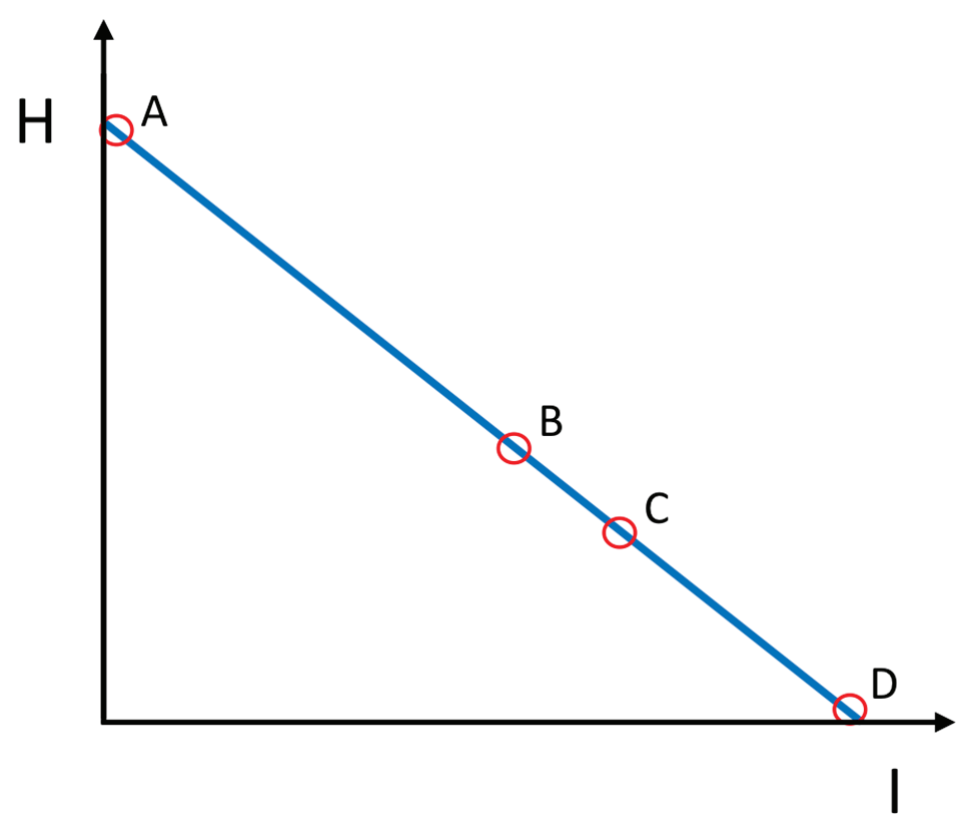
Entropy (H)-Information (I) relation in synergies. Ovals A, B, C, and D refer to respective panels on Figure 1. Oval A represents minimal agent or team integrative information (I) and maximal entropy (H). This is also the case of a maximal but non-functional uncertainty (H). Oval B is a case of the system’s increased integrative information (I) and lower entropy (H). The increase in integrative information has reduced the entropy (uncertainty), but uncertainty (H) becomes partly functional. Oval C, the integrative information (I) of the agent or team is even more increased and the entropy (H) decreased. Here, entropy (H) comes solely from the functional variability along the blue line in Figure 1. This is the ideal case of the functional integration of the agent or team, and its functional D/U potential for the external observer or the opponents is maximal. Oval D represents an excessively (non-flexibly) integrated agent or team, with maximal integrative information (I) and absence of functional D/U potential (H) for the external observer or opponents.

Concerning the entropy-information relation, the initial state of scarce co-variation between elementary variables corresponds to the case of high entropy H and low integrative information (I) between components V_1_ and V_2_ of the system (see Figure 2, oval A). As the novice learns, the synergy component variables start to co-vary, increasingly satisfying the goal constraints. Increased covariance means increased mutual information (I) among elementary variables. This gain in information is at the expense of the reduced entropy (H) of the system. A goal-attaining synergy contains large mutual (shared) information among the components V_1_ and V_2_ and low entropy intrinsic to the system (i.e., agent or team; Figure 2, ovals B and C). However, since there is a large amount of good variance, for an external observer, the synergistic system has a large uncertainty potential, while simultaneously being able to satisfy the goal constraint. This means that the synergies for an external observer are *functionally* entropic, diverse, and uncertain. “*Functionally*” means that the synergy satisfies the goal constraints. The synergy achieves the goal. For an external observer, the maximally *functional* uncertain behavior in Figure 2 would be oval C, which corresponds to Figure 1C.

Reducing maximally the variability along the blue line (Figure 1D), however, maximally increases the information and minimizes the entropy within the system as neither non-functional nor functional variability is present there (Figure 2, oval D). This can be a case for behavior in deterministic and cooperative environments in which there is only one way to attain the goal. However, for competitive and non-deterministic environments, this is quite non-adaptive behavior.

Accordingly, establishing functional couplings within the agent, agent-environment, or cooperative agent-agent system is creating integrative information or, equivalently, loss of entropy *within* the system. As the formation of such functional couplings *within* the system proceeds, the D/U potential of the system (agent or team) increases. This means that the team functionality consists of the capacity to satisfy the goal constraints in diverse ways. Because functional diversity is proportional to uncertainty (unanticipatedness), it means that the system, by becoming more diverse, becomes more *functionally* uncertain for the environment or *external observer* (e.g., opponents’ team).

It is important to see that this is valid at more levels, not only at the individual level. For example, the diversity of strategically constraining the game (different formations) will reflect the potential diversity of behavior of player dyads and individual players. Conversely, non-expert teams cannot be diversified much at the strategic level due to their lack of competencies on multiple levels. They cannot be sufficiently *functionally* diverse. In experts, in contrast, as the system becomes potentially more functionally diverse, an external observer will be less able to tell the policy, which will be used by the system to satisfy its goal constraints. The adaptive system will become increasingly functionally uncertain for the observer (or the opponent).

#### Individual Agent-Environment Level of D/U Potential

At the level of agent-environment, consider the following example: the novices’ task is to prospectively control the ball in order to drive and allocate it in the goal area. Their behavior may be very volatile and have high entropy/uncertainty within their limited space of possible action configurations. Their coupling with the ball is highly uncertain from both: their perspective and from the observers’ perspective (Figure 1A).

For example, total beginners, in their degrees-of-freedom exploratory phase of motor learning (Davids et al., 2012), will hardly successfully control the ball prospectively and each time will be surprised by the unexpected bounce of the ball off their leg. The co-ordination between their perception-action systems and the ball can be utterly uncertain from their point of view, but also from the *external observer’s* (see Figure 1A). However, this uncertainty is not functional. If the novices’ actions are uncertain and functional, then novices would be highly competitive, which is a contradiction.

In this case, there is high entropy (uncertainty) within the novice-ball system as well as high uncertainty as observed from the point of view of the external observer. There is low integrative information (I) within the novice-ball-environment system and thus high non-functional entropy (H; see Figure 2, oval A). They, the novice and the observer, can concur on the high level of non-functional uncertainty of the novice-ball system.

On the other hand, after some time of practicing, during the solution, stabilization, and especially the degrees-of-freedom exploitation phase (Davids et al., 2012), the novices’ space of possible *functional* action configurations has expanded and they can reach the goal area in different ways of controlling the ball (Figures 1B,C). Their behaviors will still be diverse and hence uncertain (entropic) from the perspective of an external observer but highly under control (non-entropic and thus predictable) from their *own* perspective (Figure 2, oval B and C). They have attained *functional* diversity and uncertainty, including, but not reduced to, deceptive movements. The ex-novice and the external observer will not concur on the degree of uncertainty or surprise of the ex-novice-ball system. For the ex-novice, ball behavior will be controllable and very predictable [high integrative information (I)], but for the external observer, it will not be predictable [sufficiently high entropy or surprisal (H)]^3^. The ex-novice will be able to control and, hence, prospectively anticipate what will happen next to the ball she/he drives (high I), but not so the external observer (sufficiently high H). In individual sports such as gymnastics, the larger D/U potential of performers produces larger surprisal, and in track and field or swimming, a larger potential set of pacing strategies and, hence, larger potential uncertainty for co-competitors (e.g., Thiel et al., 2012; Mytton et al., 2015).

#### Multi-Agent Level of D/U Potential

Teams have been depicted as superorganisms (Duarte et al., 2012). The relativity of the information-entropy principle offers a way of explaining how a group of agents becomes a team. Consider a group of novices that attempt to keep possession of the ball. Their behavior is highly uncertain, but not *functionally* uncertain. The group is, to a large degree, disorganized. The uncertainty of behavior within the group (as seen by each novice inside the group) is high, as is the uncertainty of the group as seen from outside. They do not form a unit (or units), which is (are) functional (Araújo and Davids, 2016). They do not form a team (see Figure 1A).

On the other hand, a team of trained agents is functionally uncertain in the sense that they can realize their goal (e.g., score a point or make a successful pass) in different ways by means of forming temporary task-specific units based on internal, diverse inter-agent functional couplings (Figures 1B,C). In skilled agents, affordances are used for prospective (future goal-directed) control, meaning that teammates can coordinate and form synergies more successfully, if they are well attuned to each other’s affordances (see Silva et al., 2013). A team, or within-team dyad, as a functional unit, exists to the degree in which its members contribute to the decreasing entropy (increasing integrative information) within the system and the functional uncertainty of the team for external observers (e.g., opponents^4^; Figures 1, 2). At this multi-agent level, whenever a team loses a ball due to interception by the opponent or the inaccuracy of a pass, the red oval is out of the blue line, decreasing the integrative team information (I) and increasing the non-functional entropy (H). On the contrary, when there is a successful pass, it is on the blue line and signifies the presence of integrative team information, because the combination of component variables V_1_ and V_2_ achieves the goal (i.e., satisfies the goal constraints). If the team is able to achieve the goal by passing in many different ways (combinations of V_1_ and V_2_), then its functional diversity and uncertainty as a superorganism is high. Also, the integrative team information (I) is higher than in the group of novices (Figure 2). Figures 1, 2 help in depicting the opponents’ task, which is always to push the team from state C or B toward A. In other words, to decrease the integrative information of the team and to increase internal entropy, while the goal of the team is the opposite.

On the other hand, opposing agents and teams may also co-vary. However, they do not build synergies due to the absence of the same goal, which has to be kept stable. The opponents’ goal is different and, therefore, meaningful performance goal variables are different. In the language of synergies, the opponents’ co-variance with players tends to increase the non-functionality of the team, that is, to increase the dysfunctional entropy within the opponents’ team.

### Principle 2: The Satisficing D/U Potential

Satisficing action means sufficiently satisfying behavior where the sufficiency of the outcome may be constrained by some additional criteria (Simon, 1956), for example, criteria such as dribbling past an immediate opponent, dribbling past five immediate opponents in a row, scoring a point, winning at least fifth place in an international tournament, or simply qualifying for some international championship, or winning gold medal in the world championships five times in a row. Note that the fulfillment or not of these criteria cannot be predicted beforehand but can only become clear *after the fact*. Controlling and fulfillment of such criteria would need a full model of agent or team behavior, which is currently not possible (Glazier and Davids, 2009a,b). Since the outcomes of individual movements are context-dependent and there are an infinite number of ever-changing contexts, it becomes impossible to predict the individually globally optimal behavioral pattern.

Sub-optimal behavior, on the other hand, is the one that is *sufficing* in its functionality for the given context (Byron, 1998). When speaking about the D/U potential, Simon’s concept of sufficing is close to the concept of requisite variety (Ashby, 1956). This concept describes the principle that states that in order to cope with a variable environment, the system (in our case the agent or team) has to be, at least, as variable as the environment. By joining the two principles, one can speak of *sufficing variability*, that is, variability that suffices for attaining the goal (Hristovski, 2017). For example, players facing an undefended space during a counterattack (i.e., having a large D/U potential) would typically use small behavioral variability (i.e., small D/U behavior), which is sufficient to conquer the space as quickly as possible and try to score a point. On the other hand, if they are approached by one or two defender players, which reduce their D/U potential, they will increase behavioral variability (i.e., the D/U behavior) to some level of sufficing in order to achieve the goal. The achievement or non-achievement of the goal will *post factum* tell whether the level of behavioral variability was sufficing. In general, whereas in stable and cooperative environments the tendency of behavior is to attain low action entropy potential – stabilizing tendency (e.g., walking on flat surfaces such as streets), in uncertain and non-cooperative environments, adaptive behavior is to sufficiently increase the action entropy potential in order to be able to satisfy the goal constraints of the organism. Learning to detect the level to which the D/U potential has to be engaged depending on the opponent is of utmost importance for the success of athlete and team performance. Specific training methodologies may be needed to develop this aspect of abilities. This process is based on permanent co-adaptation of the agent/team-environment/opponent system that sets the asymptotic level of convergence.

**Figure 3:**
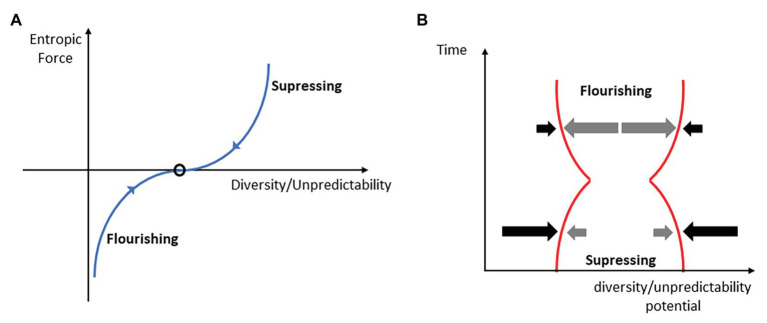
The system is subject to forces that tend to suppress the D/U potential. (A): the balance of suppressing (opponent) and flourishing entropic forces (gradients) determines the D/U potential value (black oval) of the behavior. We hypothesize that the larger the deviation from the average diversity/uncertainty of behavior (the oval), the larger the suppressing or flourishing entropic force will be. (B): cooperative-competitive intelligence (CCI) manifests as the ability to act prospectively. After a narrowing down of the D/U potential as a result of the suppressing forces of opponents, the tendency is to be compensated *at least* to the initial level (*time* = 0). This is a consequence of flourishing forces (see A).

Wissner-Gross and Freer (2013) describe intelligence as future entropy maximization tendency. However, in biological systems, global entropy maximization may have limits due to the energy costs of such a behavior. A good example of such restriction in biological systems is the overcompensation phenomenon. It can be detected at cellular, functional, or overt performance level after a suitable amount of perturbation (training impulse) applied to the agent. Overcompensation is the evanescent state of increased functional (integrated information) potential of the organism, which vanishes if the organism is not faced with certain continuity of such perturbations. In the introduction, we defined the functional action potential as a D/U potential. Hence, due to the perturbation, i.e., fatigue, the diversity potential of the organism temporarily decreases, and the cell or organism reacts *prospectively* by a temporary increase in the diversity (integrated information) potential. It most likely anticipates^5^, in a sense of strong anticipation (Dubois, 2003; Stepp and Turvey, 2010), the possible incoming perturbations and prepares to negotiate them with enhanced potential. This is a clear example of intelligent behavior. However, the biological system does not continue to increase the potential without limits, although there are immediate available excess resources for it that can be used (e.g., glycogen deposits). The satisficing principle is due in part to the fact that each agent has limited resources of energy for action and for *globally* maximizing the space of future action possibilities, i.e., the D/U potential, would quickly exhaust energy resources. There seems to be a trade-off of energy and entropy/information properties. If perturbations cease, the D/U potential returns to the pre-perturbation level, which it temporarily decreases. This phenomenon has been routinely detected on a macroscopic measurement scale as a daily or monthly (Verkhoshansky and Siff, 2009) time series of ability performances (see Figure 3).

Here, it is important to note that the non-decreasing D/U potential is obtained within a certain temporal window, temporal prospect, or horizon. It will continue to increase only if the environment applies a perturbation to the system, by temporarily suppressing the D/U potential. Otherwise, it remains adapted by sufficing the D/U principle to the current environmental demand. The temporary suppressing of the D/U potential, as, for example, getting fatigued during the training session, is made to prospectively increase it, which is the CCI goal within a certain temporal frame. On the contrary, excessive perturbations may cause long-term suppression of the potential, which is not the CCI goal in the said temporal frame.

Thus, it seems plausible to claim that the long-term environmental requirement of sufficing D/U potential in agents and teams *is the reason* for the emergence and evolution of properties such as degeneracy, pluripotentiality (Seifert et al., 2013), metastability (Kelso, 2012), and synergies (Latash, 2008), which, in a circular causality fashion, stabilize the development of the D/U potential. In this light, it seems that the striving toward the non-decreasing action space of possibilities is why biological degeneracy, pleiotropy, and metastability have been evolutionarily stabilized. While degeneracy and pleiotropy (pluripotentiality) form the basis of the repertoire (set of synergies) of individual or team actions (Seifert et al., 2013; Ric et al., 2016), the dynamic mechanism of metastability is responsible for switching among them (Hristovski et al., 2009; Kelso, 2012; Bruineberg and Rietveld, 2014). The net result of the non-decreasing entropy tendency in agents and teams is attaining stability through flexibility and stability of the flexibility (defined as a part of the diversity).

Hence, the satisficing principle as a sub-optimal solution can be expressed as subject to satisfying inequality constraints (e.g., Δ*H* ≥ 0), which means that the satisficing solution for any agent or team is any *average* value of *ΔH* (change of D/U potential) that is equal to or greater than 0. This means that CCI can be conceptualized as an average behavioral tendency of agents and teams to resist their future actions to be suffocated by the environmental (opponents’) perturbations. In this formulation, the upper limit of behavioral diversity/uncertainty H is determined by the satisficing principle. The average here exists because, on some occasions (as in the case of overcompensation), in absence of perturbations, the D/U potential may converge to some arbitrarily given initial value after passing the period of increased potential, which will mean its local decrease. The decrease is, in fact, a re-adaptation to smaller environmental challenges (perturbations). This, however, adds another property to the CCI. In order to grow on average, more often than not, the agent or team must be subject to perturbations that will stimulate the fulfillment of CCI growth conditions. Hence, the satisficing principle puts demands on the system that needs to grow its CCI. Growth of intelligence needs perturbations in the other direction to H growth, to which the system will respond with Δ*H* > 0 behavior. In other words, this means that the development of CCI is necessarily dependent on environmental dynamic properties. A system coupled to challenging and stimulating environments represents a system of growing CCI. The term “challenging” here means perturbations that are strong enough to provoke the growth and evade the temporary stalemate, which may, on a longer time scale, turn into a decrease in the D/U potential.

### Principle 3: The Prospective Non-decreasing Action D/U Potential

This principle claims that a system (agent or team) tends toward non-decreasing D/U potential: the system develops a reactive force in an opposite direction to any perturbation from the environment/opponents, which reduces the previous state of D/U potential (Hristovski, 2017). What the opponent strives to do is to minimize the D/U potential of the opponent or team and simultaneously increase or at least maintain its own satisficing level of potential action entropy.

At the basic level, two forces are molding the behavior: nested goal gradients and the entropy (decrease-increase) force. These forces drive the adaptive response of the system. The nested goals may be keeping the ball in possession, in order to be able to score a point, in order to win (survive) the match. This “nestedness” already assumes a level of D/U potential that can attain these sub-goals in the face of permanent perturbations of the opponent to reduce the chances of attaining the goals by reducing the D/U potential. On the other hand, the force toward non-decreasing D/U potential can in fact be defined as a goal-setting force. The system’s general goal is always not to allow the decrease of its opportunities for action. Seen in a certain time frame, this means that, even if the system is temporarily pinned down and has reduced prospective action D/U potential, it always seeks ways to escape from this state. Moreover, in competitive environments, CCI would seek to escape from this state with a sufficing rate, given the constraints. This can be expressed as the sufficing entropy rate or production. For example, a player that has to temporarily dribble-pass one or a few opponent players, through a reduced D/U potential corridor, will negotiate this situation in sufficing rate by having in prospect the perceived free space (larger D/U potential) located further in the field. Otherwise, if the situation affords her/him not to succeed in this action, s/he may decide to pass the ball to a teammate, which sufficiently increases the D/U potential of the team prospectively. This is the same *prospective* adaptive reaction that we described earlier in relation to overcompensation. The reactive force simply acts as a negative feedback, not allowing the initially reduced D/U potential to become even more reduced in the future, so the system becomes pinned down to some minimum, which would most likely bring about non-achievement of the goal (e.g., a stolen ball, receiving a goal, losing the match, etc.). Hence, the larger the *accessible* time scale at which the system non-decreases its entropy, the larger its CCI. Being sensitive to constraints that play a crucial role at these time scales is a part of intelligent behavior. In general, the larger the time frame^6^ of the prospective action D/U potential (H), and the quicker one escapes from its reduced state, the larger the CCI behavior.

More generally, the co-adaptivity at multiple time scales and levels may be defined as a competition of two forces: (a) the tendency to decrease the opponent’s opportunities of action and increase their informativeness or predictability (suppression) and (b) the tendency to increase one’s own D/U potential, i.e., flourishing (see Figure 3).

In fact, in the light of the interplay of these forces, opponents and fatigue (on different time scales) play identical positive adaptive roles, temporarily reducing the potential prospective D/U potential, while pushing the agent’s organic systems, or the team, to recover or overcompensate. This non-decreasing entropy tendency is basically an *anti-fragility* phenomenon (Kiefer et al., 2018), claimed as a general principle in sociology, psychology, and biology from cell to society (Taleb and Douady, 2013).

Some consequences for the emergence of competitive teams stem from these principles. In general, non-decreasing D/U potential, at the level of agent or group of agents, is often only possible through social cooperation. In sports teams, social cooperation is underpinned by becoming sensitive to affordances. Thus, at performance level, becoming attuned to each other’s affordances (Silva et al., 2013) is one of the *means* through which the principle is realized. In other words, the tendency of non-decreasing D/U potential is the *driving force* of the formation and stability of social structures in general and of sports teams in particular. That is, the formation of social structures seems to be a consequence of CCI. As we mentioned above, teams exist to the degree to which their members contribute to their non-decreasing D/U potential. From this perspective, intelligent behaviors, individual, dyadic, and collective, simply emerge as a consequence of satisfying non-decreasing D/U potential constraints.

Within the framework of ecological dynamics, decisions are grounded in actions (Araújo et al., 2006, 2019). Actions are agents’ decisions. The actions of living agents are always future-oriented, i.e., prospective (Turvey, 1992), based on perceiving opportunities of action (affordances) that the environment offers and which are not only immediate but also more distant in time (Bruineberg and Rietveld, 2014; Seifert et al., 2014). Hence, CCI at performance level is fully embodied because it is not only crucially dependent on, but also to a degree consists of, bodily capacities (effectivities), which make the usable set of affordances larger, and hence, more flexible. CCI is the capacity of (individual and collective) decision-making to always non-decrease (i.e., maintain or increase) the *prospective* D/U potential. In other words, CCI is a tendency to keep at or grow to, the satisficing level of the prospective control of behavior within some time horizon^7^. Creativity is one of the means to grow the D/U potential.

The game is a permanent exploration of possibilities for satisfying the sub-goal of scoring a point and consequently the main aim of winning the game, i.e., to survive. In this sense, the exploratory phase can be considered as an “incubation” period of the creative process, before the sudden emergence of the satisficing solution that leads to the scoring point (i.e., the satisfaction of the goal constraint). Whenever, the environment (opponent) temporarily suppresses the D/U action potential, the agent (individual or team) is constrained to find/create a solution to the immediate circumstances in order to recover its previous D/U state of possibilities or increase it (Hristovski et al., 2011). After a perturbation that decreases the D/U potential, the system strives to compensate or over compensate the previous potential level of action entropy. Flourishing is a process/state, characterized by an increase in the D/U potential action entropy and is based on creativity. Suppressing habitual action policy and discovering a new mode for attaining the goal is a mode of creativity, (e.g., Torrents et al., 2020). If CCI can be defined as a tendency of non-decreasing the D/U potential, then it follows that there is a tendency of (intrapersonal and inter-personal) positioning in the zone from which a large set of actions are easily achievable (switchable), that is, the zone of optimal grip on the field of affordances (Bruineberg and Rietveld, 2014).

Hence, with respect to the definition of the CCI as “finding the best solution” (Memmert et al., 2010; Memmert, 2015), from the aforementioned, it follows that only if the agent or team is not pinned down (i.e., she/he has a satisficingly large solution space in prospect), can s/he detect the sub-optimal solution (Byron, 1998) in a form of acting on affordances that sufficiently satisfies the task goal constraint. If initially cornered, then a better solution will be the one that will open his/her space of opportunities (enlarging the D/U potential). The non-decreasing D/U potential is, in fact, the *goal* of every CCI system. The game theory definition of game intelligence (Lennartsson et al., 2015) is the probability of the offense scoring the next goal minus the probability that the next goal is scored by the defense. This definition directly follows from the principle we are currently discussing. Only an agent or team with, on average, larger D/U potential can have a larger probability of scoring a point than the opponents. Note that this is valid not only for phases of ball possession but also for phases of defense. For example, a “bunker defense” in football may effectively suppress the opponents’ attacking D/U potential (see Figure 3) by keeping opponents further from the goal area and lowering the probability of scoring a point. It also increases the undefended opponents’ space on the pitch (increased D/U potential for counter-attack). One can see that both definitions of game intelligence can be inferred as special cases of the non-decreasing D/U potential principle.

In fact, the trade-off of suppressing and flourishing (Figure 3) signifies the interplay between one type of creativity (see Torrents et al., 2020) and the CCI. Oftentimes, creativity is fostered when the environment does not enhance the opportunities of action of the agent (performer or team), but instead suppresses them (Hristovski et al., 2011). This occurs when the environment (opponent) does not subside to perturbations by the agent or the team. It occurs when there is a *negative feedback* from the environment as a response to the agent’s actions. While co-adaptivity within the team strives to produce a *positive feedback* for some initial possibility enhancement, co-adaptivity between opponent teams strives to create a *negative feedback* that tends to suppress the initial enhancement of action entropy in the opponent’s team. Hence, CCI may be related to creativity to a degree, which, in fact, has been demonstrated in recent studies (Memmert, 2015).

#### Biological Intelligence as a Non-decreasing D/U Potential

We have already considered biological overcompensation as a fundamental expression of biological intelligence that satisfies our conceptualization of CCI. However, the suppressing-flourishing dynamics of D/U potential can also be particularly well detected in various forms of reciprocal compensations between psychomotor dimensions during the agent’s or team’s action. Psychomotor variables such as agility, power, strength, accuracy, speed, endurance, timing resolution, etc., as well as morphological variables (Hristovski and Dukovski, 1996) are self-organizing^8^ properties of the agent-environment system (Hristovski et al., 2010; Hristovski, 2017). In ecological psychology, on the one hand affordances and on the other motor abilities and morphological variables (i.e., effectivities) are complementary to each other. Affordances are body‐ or action-scaled (Fajen et al., 2008). For example, the endurance ability directly enables a larger diversity of immediate or time sequences of affordances, i.e., potential running tactics.

These variables and their interactions are part of what may be called biological intelligence. The larger the volume within the effectivities space, the larger the field of affordances on which it can be acted (Bruineberg and Rietveld, 2014), given the rest of constraints.

**Figure 4:**
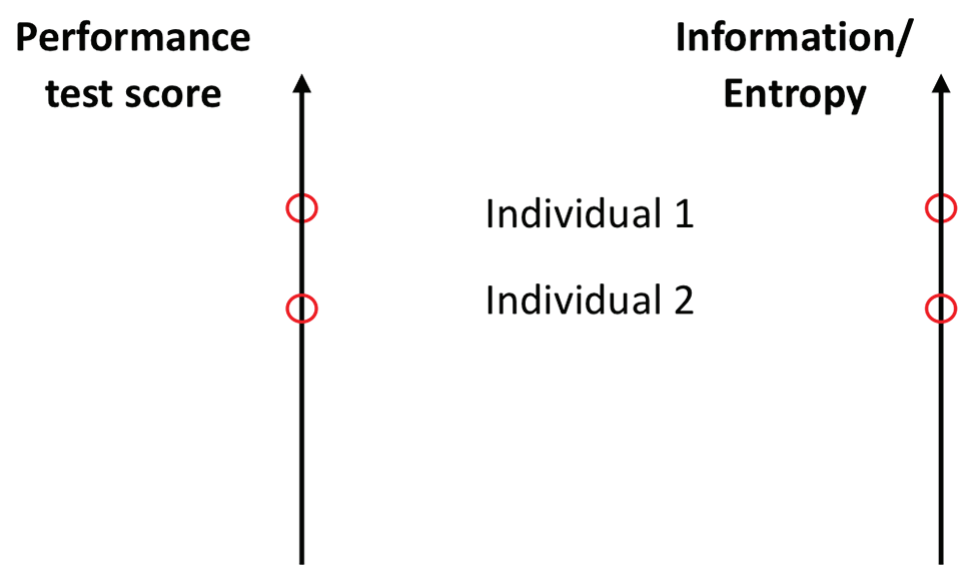
Two interpretations of measurement scales. **Left panel**: the traditional interpretation: individual 1 shows a larger performance or score than individual 2 in certain ability or morphology tests. **Right panel**: individual 1 exhibits larger entropy/information in the same ability test. Here, entropy/integrative information is measured as a logarithm of the length of the scale interval to the position of the oval. One can immediately generalize this definition to n-dimensional space (volume) spanned by n-independent measures of abilities. In a first approximation, the entropy/information will be the sum of all of them.

Performance in all of these variables depends on the effectivity of coordinative processes at many levels starting from the cellular metabolic to the organism-environment level. Bosch (2015) discusses this from the aspect of intra‐ and inter-limb coordination. What we understand here as coordination subsumes Bosch’s ideas, but also coordination among all levels. For example, the synchronization of motor units is a type of intramuscular coordination. In addition, co-adaptation between cardiorespiratory systems (Balagué et al., 2016; Garcia-Retortillo et al., 2019) is coordination, although measured at the physiological level. This does not mean that the coordination at this level is independent. On the contrary, it is constrained from below and from above (Balagué et al., 2019). Hence, a larger performance in any or all of these abilities means a larger D/U potential of coordinative patterns and, hence, can be measured as entropy variables (Hristovski, 1989; see Figure 4). An agent with a larger strength or power has excess potential of coordinative configurations, and hence, a larger D/U potential. Also, his/her integrative information (I) is larger, signifying a larger number and better reciprocal compensatory couplings among the components of the system. Hence, the D/U potential of coordinated components is larger.

It is important to emphasize, however, that all these separate abilities are possibly always contextualized within a certain form of life (Rietveld and Kiverstein, 2014) and molded by each professional environment (e.g., sports discipline). As lab-tested abilities, they are often not representative of their contextualized action manifestations. However, what was said above is valid also for the more contextualized variables once they become measurable. Therefore, we hypothesize that these abilities also form mutually compensatory and co-varying (i.e., dimension reducing) synergetic sets of variables *specific* for each sports discipline and are specific to individual agent-environment systems. These entropy synergies arise as a consequence of the suppressing-flourishing dynamics of D/U potential and would be a part of what we call here biological intelligence.

For example, the possible synergies (reciprocal compensations) between attention focus and body acceleration may be investigated. In certain contexts, the lower acceleration ability of an agent may be compensated by his/her larger attention focus and acuity, or vice versa. Another example on an interpersonal level concerns the interplay between morphological and motor ability compensatory interplay. In order to decrease the D/U potential of the opponent, a boxer with long arms may keep the opponent with shorter arm length at a distance. Here, the length of the arms means larger/smaller potential space control, and hence, larger/smaller entropy, i.e., D/U potential (suppression/flourishing in the morphological/spatial dimension). The latter will have to reciprocally compensate his/her shorter arms disadvantage and his lowered D/U potential by an increased degree of agility performance (flourishing in agility dimension) and attempt with short unanticipated incursions to satisfy her/his goal constraints. The third example concerns the action direction (directional D/U potential) of a player and the free space (D/U) available toward the goal area: a player that has vast space available toward the goal (large D/U potential) typically moves by the shortest path (low D/U). However, when opponent players try to reduce her/his space of action potentialities (i.e., to lower her/his D/U potential) s/he switches to higher entropic (higher D/U) behavior in an attempt to dribble-pass opponents.

An intelligent response of agents to the reduction of the D/U potential in a certain dimension tends to be compensated by a future prospective increase in D/U behavior in the same or another dimension or dimensions. This reciprocal compensatory co-variation of different ability dimension entropies is, hypothetically, an instant of intelligent behavior, present also on a social (team) level. For example, a quick reallocation of the ball can be achieved not only by a very fast player but also by a well-synergized team of moderately fast players.

## Archetypical Motifs of CCI Dynamics

The CCI theory enables not only scientific but also some more qualitative, philosophical research directions. In his paper “Sport as a drama” (Kreft, 2012), the author states that dramatic aspects of sport and sports games are more existentially dense and aesthetically attractive than theater dramatics since the actors are real persons, taking real risks (p. 230). Concurring with Kreft, we would like to briefly comment on how the CCI theory, particularly suppression-flourishing dynamics, captures the dramatics of cooperative-competitive events (e.g., sports, games, and life itself), by containing deeply archetypical aspects of the existential striving of human beings and living forms. In sport, as in any drama, one can readily detect most, if not all, elements of Freytag’s dramatic structure (Freytag and MacEwan, 1908) such as exposition, rising action, climax, falling action, and catastrophe. As previously explained, from the aspect of CCI theory, in sports, these elements emerge spontaneously at many time scales as a result of the antagonistic action of two entropic forces, namely, the flourishing and suppressing force. In our view for the philosophy, especially the aesthetics, of sport, it would be important to analyze the content of antagonistic archetypal motifs such as: Eros vs. Thanatos, survival vs. extinction, hope vs. despair, life vs. death, freedom vs. confinement, etc. These qualitative aspects stemming from the CCI theory may be the core of Kreft’s existential density that forms the dramatics of sports competition. On the other hand, these possibly pertinent relations between quantitative entropic forces and the qualitative experience of antagonistic motifs may enable a fruitful realm of future mixed-methods research in sports philosophy, sociology, and psychology. An example of this kind of research could be the relationship between the behavioral and archetypal experiential dynamics of athletes and supporters during phases of dominant suppression and flourishing quantified as D/U potential.

## An Outline of a Further Research Program

### Theory Predictions and Testing

A desideratum for any scientific theory, aside from its conceptualizing and explanatory power, is to be able to make predictions about the behavior of the system it deals with. At the level of sports performance, the theory of CCI puts forward a general prediction (hypothesis) that can be formulated in the following way: all actions of the system (individual agent or team) emerge from the interplay of two forces subject to specific constraints: the entropic forces as explained above and the general goal of the system. In the text that follows, we offer two interacting strategic approaches in order to test this general prediction. Specific models and predictions (hypotheses) that stem from it can be formulated as behavioral scenarios. We also provide some examples of these scenarios.

### Theoretical Modeling Deductive Approach

Sports behavior stems from a multidimensional dynamic system with cooperative and competitive interactions. A suitable deductive approach to understanding CCI would consist of building agent-based models (see Bonabeau, 2002). However, instead of the usual practice of providing each agent with a set of specific rules for each dimension, and scenario-dependent rules of behavior, initially agents can be constrained in fewer dimensions by the principles depicted above. For example, the motivation climate (e.g., Duda and Appleton, 2016) as a slowly changing variable (Balagué et al., 2019) may be applied to an individual agent-environment (Withagen, 2018) and/or at the team level as the entropy parameter. The cascade effects toward quicker processes may then be simulated down to performance level. Entropy principles, which could be applied as a “common currency” among simulated dimensions of behavior, seem more parsimonious due to the substantial reduction of the simulation cost. However, the main advantage and heuristic strength of this approach would be that, in such a scenario, predicted behavioral rules will emerge out of the interaction between the principles and the contextual constraints. For example, we saw previously that space conquering or creation, passing, dribble-passing, or invading actions can be predicted as being a consequence of the tendency of non-decreasing D/U potential. The first step would provide basic behaviors that can be simulated at the level of dyads and increasingly higher-order collectives for different scenarios. These basic predicted emergent behaviors would be the outcome of a small, partial, subset of the full set of dimensions present in real-world behaviors. The level of fidelity of these behaviors can then be validated by comparing the essential variables extracted from the simulated behavior with real-world data from identical scenarios. Based on the detected similarities and differences between simulations and real-world data, the next step would be the parametrization of the model by additional dimensions, i.e., constraints, which can also be cast in the form of satisfaction conditions of the same principles. In this iterative process, we suggest that one can finally reach high-fidelity simulations of cooperative-competitive intelligent behavior and then, by manipulating certain constraints, study the quantitative and qualitative changes of behavior. For example, intelligence defined as a tendency toward non-decreasing D/U potential may be at odds with ethics. In order to maintain or increase its options, the system may not act in coherence with some basic social values. This can be an interesting topic for future research. In this way, one can hope to genuinely understand such cooperative-competitive behaviors based on a few basic principles.

### Empirical Inductive Approach

The empirical research could proceed in parallel to the deductive approach outlined above. As we saw in the text above, CCI manifests as a tendency toward non-decreasing D/U potential. Note that it is the “common currency,” that is, entropy formulation of D/U potential, which can reveal the compensatory processes of the intelligent coping of systems. As described in the previous text, oftentimes, the suppressed D/U potential in one dimension may be compensated in another dimension and enable flourishing. If we analyze the compensatory processes in their manifest dimensions (space, direction, number of possible passes, agility, etc.), we will hardly be able to detect and formulate the existence of compensatory phenomena in the space of D/U capacity. Some examples of research may include the following elementary scenarios (predictions): (1) If a certain space becomes occupied by opponent players (reducing the behavioral D/U potential of the team), a teammate demarks another spatial position in order to receive the ball (increasing the team’s behavioral D/U potential). In this case, the dimension state of numerical imbalance is transferred to a relevant spatial dimension state, and both can be formulated in entropy units. (2) The team synchronously moves into the opponent’s half of the field. Synchronization displays low entropy behavior in the direction or relative phase dimensions. However, the D/U potential of passes between teammates increases because of the synchronous movement of the team centroid to the opponents’ half. Where there are more teammates, there are more possible passes and, in general, larger D/U potential. In this case, the directional (or relative phase) dimension state is transferred into the connectivity (number of possible passes) state dimension, and both can be formulated in entropy units. (3) Another scenario is when the occupation of space by opponents would correspond to a perturbation that lowers the team’s action space and a teammate’s demarcation to the recovery compensation corresponding to increasing opportunities for action. In this case, the possibility of spatially defined (or numerical imbalance) lost action transfers into an action possibility gain of the angular dimension, and both can be formulated in entropy units. (4) As a result of conquered space by opponents, the player that possesses the ball compensates his lost space by increasing her/his entropy of actions (attempts to dribble-pass the opponents who have conquered the space). In this case, there is compensatory behavior from the spatial dimension into the individual action dimension. Both can be formulated in entropy units.

Other scenarios of such compensating synergic phenomena may also be predicted as multidimensional, spontaneously emergent, compensatory cooperative-competitive, intelligent behaviors. The results of these and similar studies would have mutually supporting and modifying interactions with results coming from the deductive approach. Big data analytical tools (multilayer neural networks, support vector machines, deep learning, and other current and future analytical techniques) coming from Machine Learning Toolbox, may be used to extract the essential macroscopic, mesoscopic, and microscopic behavioral variables. These variables can then be used in a circular regulative manner for improving the deductive modeling part of the research. In this fashion, an original and fertile research program can emerge in the future.

## Concluding Remarks and Practical Aspects of the Theory

The full-blown detailed practical consequences of the theory can be assessed after sufficient research with content as described in the previous section. However, some preliminary notes on the practical work of coaches and athletes, or participants in cooperative-competitive activities in general, can be outlined. In the theory, CCI was conceptualized as the capacity of an agent or a team to successfully *evade* or *escape* the state of reduced sufficing D/U potential quickly enough. To successfully evade the reduction in D/U potential, one has to be able to prospectively negotiate environmental perturbations. To successfully escape, one has to develop the multidimensional transfer of D/U potential in the form of multidimensional synergy reorganization. Some examples for practitioners to consider are given below.

The theory of CCI fosters the development of capacity measures for escaping and evading the reduced D/U potential. At the individual CCI level, self-reliant agents (e.g., players) who are not dependent on detailed instructions of coaches may be able to unleash larger D/U potential. Hence, skill acquisition pedagogies, which promote this kind of “hands-off” approach, seem to have a greater potential for accomplishing this task (Davids et al., 2008; Chow et al., 2015; Button et al., 2020).

Pol et al. (2020) argued that the objective of the training process itself should be rethought: instead of excessively focusing on the decontextualized development of the conditional, motor, and psychological attributes or dimensions separately, work should be done on developing the context-dependent D/U potential of athletes/teams. As argued previously, the development of the D/U potential may emphasize, specific to each sport contextualization, the functional reciprocal compensations (i.e., synergies) among different dimensions (e.g., motor, conditional, psycho-affective, collective, and social). For instance, anxiety, injury, or stress in one player, which effectively reduces his/her D/U potential, can be compensated through strategic collective tactical actions prescribed by the coach. However, the work on the skills of athletes/teams to functionally self-reorganize, within the ethical^9^ norms, by finding fast multidimensional compensations would be a worthwhile long-term endeavor.

This aim also includes the development of pluripotential (i.e., pleiotropic) players in collective sports (Rangel et al., 2019), that is, players with *sufficiently* overlapping tactical roles. The development of sufficiently pluripotential players and teams involves work on the following sub-tasks: (i) practitioners and researchers should work jointly on determining the degree of skills and competencies overlap in the team, which is satisficing and contextualized for different types and intensities of perturbations by the opponents, (ii) working on the way in which D/U potential-reducing perturbations of different types may be dampened. For example, it can be achieved in the form of task redistribution within sub-groups of players with overlapping competencies, (iii) acquiring skills on negotiating characteristic channels within the team through which D/U-suppressing perturbations are spread by different types of opponents and perturbations, (iv) managing to negotiate the formation of characteristic “hot or task congestion spots” within the team and their characteristics for different types of opponents and perturbations, and (v) learning how to dampen further perturbations across a team in order to eliminate the decreased D/U potential hot spots or to reduce the likelihood of their formation.

In order to develop the athlete/team D/U potential, critical training zones (or *zones of abundance*) may be detected by experienced coaches through the manipulation of constraints (Hristovski et al., 2013). These zones are characterized by the locally maximized D/U potential of the athlete/team. Practicing in this kind of zones may provide a boost to the necessary perception-action skills of athletes.

CCI theory also suggests that working on skills for quick detection and adaptation of the satisficing D/U potential, relative to the opponents, is of key importance during training and competition. In competition, athletes and coaches often make strategic assessment of the requisite use of resources for every opponent. Objectively, more competitive athletes/teams may lose against less competitive ones because the latter often increase their D/U potential when competing against superior opponents. The often-ignored reduced anxiety and increased motivation of inferior athletes/teams when competing with more powerful adversaries should be carefully considered since it increases their D/U potential. In contrast, the lack of motivation when competing against inferior opponents may reduce the D/U of highly competitive teams too much, leading to unexpected results (Clancy et al., 2016).

The CCI theory elaborated in this paper may also prove to have strong integrative capacity since it may be used to channel the practice in relatively disparate domains of human activities. For example, in the domain of well-being, diversification through compensatory activities in different domains (i.e., dimensions) other than stressful professional ones shows an increase in well-being experiences (e.g., Conner et al., 2018). In the realm of health, activities that increase the multidimensional D/U potential and compensatory synergies may prove to be of great importance (Balagué et al., 2020). According to the theory developed in this paper, practical work on these and similar issues supports the growth of CCI in the areas of sports, health, and well-being in physical activities.

## Data Availability Statement

The original contributions presented in the study are included in the article/supplementary material, further inquiries can be directed to the corresponding author.

## Author Contributions

RH conceived and wrote the manuscript. NB reviewed the previous versions. All authors contributed to the article and approved the submitted version.

### Conflict of Interest

The authors declare that the research was conducted in the absence of any commercial or financial relationships that could be construed as a potential conflict of interest.

## Footnotes

^1^Information as a quantity here should be understood as arising from co-varying variables or processes and as a magnitude of integration or organization (e.g., Haken, 2006) rather than in the sense of capacity of communication channels or codes (Shannon and Weaver, 1949). On the other hand, in the text that follows, ecological information will be named as such.

^2^It may be a dilemma why we use the term uncertainty and not degeneracy, for example. While uncertainty encompasses degeneracy, which is the capacity to attain *similar outcomes* by the spatio-temporal arrangements of structurally different components (e.g., Edelman and Gally, 2001), the D/U potential may also include successful changing of goals and intended outcomes. Also, it can include attaining different outcomes by the same means (see Pol et al., 2020). In this sense, the D/U potential of behavior has a wider meaning than degeneracy alone. Also, importantly, uncertainty is a *relational* variable that exists only at the interface between the performer and the environment (e.g., opponents), while degeneracy is a property of the agent or team.

^3^They can concur on the level of functional diversity, though. Both, the observer and the ex-novice will judge the functional diversity as increased.

^4^With respect to this, one can make a distinction between the level of adapted *skills* and *expertise* and the level of cooperative-competitive *intelligent behavior*. An agent can have a high level of skill and expertise but, contextualized by some personal and social constraints, could act in a way that decreases the level of the D/U potential of the team. Some trivial examples are as follows: an expert player that is perseverant in unsuccessful solo actions driven by excessive self-interest or an expert player that commits a fault driven by revenge. In a similar vein, some social and personal constraints may be in conflict with the tendency of non-decreasing diversity of prospective control, e.g., some ethical constraints. This would limit the greedy tendency towards *maximizing* the D/U potential. Hence, because of this, cooperative–competitive intelligent behavior *is not* only, or at least *not dominantly*, based solely on cognitive processes.

^5^One can hypothesize that overcompensation is an evolutionarily stabilized instantiation of strong anticipation, a purely dynamic effect in which the “slave” system coupled to its “master” system anticipates the behavior of the latter. In this case, the master system would be the environment and the slave, the cell/agent.

^6^The time frame at the level of performance can be quite short due to the nondeterministic dynamic environment. However, at the level of strategic planning, which includes, for instance, performance analysis of the opponents, it may be much longer. These different timescales, however, cannot be compared with respect to CCI. On the other hand, they are nested and are probably subject to circular causality (Balagué et al., 2019).

^7^The time horizon at the level of performance is orders of magnitude shorter than the one characteristic for the strategic planning of matches, etc. However, the principle is valid for all these temporally nested activities.

^8^The existence of performance fluctuations in all these abilities, or in general what is traditionally referred to as “psychomotor traits” (see Delignières et al., 2004), is sufficient evidence of their soft assembly. At each trial, component processes are coordinated (assembled) more or less differently. Performance fluctuations are an inevitable consequence because they are assembled online each time. There is no “ready-to-use”, fixed in detail, pre-formed, dormant potential inside the person or team that can be merely “activated” in its unchanged shape. On the contrary, the functionality and reliability of such dormant, “ready-to-use” structures would have near-zero fluctuations similar to the execution of computer programs.

^9^Some unethical (or borderline) examples of behavior aimed at reducing the D/U potential of opponents are as follows: losing time feigning injury or holding back the ball under constraints of winning.

## References

[ref1] Araújo D. Davids K. (2016). Team synergies in sport: theory and measures. Front. Psychol. 7:1449. 10.3389/fpsyg.2016.01449, PMID: 27708609 PMC5030782

[ref2] Araujo D. Davids K. Hristovski R. (2006). The ecological dynamics of decision making in sport. Psychol. Sport Exerc. 7, 653–676. 10.1016/j.psychsport.2006.07.002

[ref3] Araújo D. Hristovski R. Seifert L. Carvalho J. Davids K. (2019). Ecological cognition: expert decision-making behaviour in sport. Int. Rev. Sport Exerc. Psychol. 12, 1–25. 10.1080/1750984X.2017.1349826

[ref4] Ashby W. R. (1956). An introduction to cybernetics. London: Chapman & Hall.

[ref5] Balagué N. Almarcha M. C. Hristovski R. (2020). Updating exercise prescription in health and disease. Phys. Ed. Sport Health 9, 3–6. 10.46733/PESH2036pg

[ref6] Balagué N. González J. Javierre C. Niño O. Alamo J. Aragonés D.. (2016). Cardiorespiratory coordination after training and detraining. Principal component analysis approach. Front. Physiol. 7:35. 10.3389/fphys.2016.00035, PMID: 26903884 PMC4751338

[ref7] Balagué N. Pol R. Hristovski R. Torrents C. Ric A. (2019). On the relatedness and nestedness of constraints. Sports Med. Open 5:6. 10.1186/s40798-019-0178-z, PMID: 30742241 PMC6370894

[ref8] Balesku R. (1975). Equilibrium and non-equilibrium statistical mechanics. New York: Wiley, 1105.

[ref9] Black D. R. Riley M. A. McCord C. K. (2007). Synergies in intra-and interpersonal interlimb rhythmic coordination. Motor Control 11, 348–373. 10.1123/mcj.11.4.348, PMID: 18042965

[ref10] Blue K. (2009). Smart golf: an exploratory study of sport intelligence in golf. doctoral dissertation. Michigan State University.

[ref11] Bonabeau E. (2002). Agent-based modeling: methods and techniques for simulating human systems. Proc. Natl. Acad. Sci. U. S. A. 99(Suppl. 3), 7280–7287. 10.1073/pnas.082080899, PMID: 12011407 PMC128598

[ref12] Bosch F. (2015). Strength training and coordination: An integrative approach. Netherlands: 2010 Publishers.

[ref13] Bruineberg J. Rietveld E. (2014). Self-organization, free energy minimization, and optimal grip on a field of affordances. Front. Hum. Neurosci. 8:599. 10.3389/fnhum.2014.00599, PMID: 25161615 PMC4130179

[ref14] Button C. Seifert L. Chow J. Y. Davids K. Araujo D. (2020). Dynamics of skill acquisition: An ecological dynamics approach. Champaign. IL: Human Kinetics Publishers.

[ref15] Byron M. (1998). Satisficing and optimality. Ethics 109, 67–93. 10.1086/233874

[ref16] Castellano J. Fernández E. Echeazarra I. Barreira D. Garganta J. (2017). Influence of pitch length on inter‐ and intra-team behaviors in youth soccer. An. Psycol. 33, 486–496. 10.6018/analesps.33.3.271051

[ref17] Chow J. Y. Davids K. Button C. Renshaw I. (2015). Nonlinear pedagogy in skill acquisition: An introduction. London: Routledge.

[ref18] Clancy R. B. Herring M. P. MacIntyre T. E. Campbell M. J. (2016). A review of competitive sport motivation research. Psychol. Sport Exerc. 27, 232–242. 10.1016/j.psychsport.2016.09.003

[ref19] Conner T. S. DeYoung C. G. Silvia P. J. (2018). Everyday creative activity as a path to flourishing. J. Posit. Psychol. 13, 181–189. 10.1080/17439760.2016.1257049

[ref20] Couceiro M. S. Clemente F. M. Martins F. M. L. Machado J. A. T. (2014). Dynamical stability and predictability of football players: the study of one match. Entropy 16, 645–674. 10.3390/e16020645

[ref21] Davids K. Araújo D. Hristovski R. Passos P. Chow J. Y. (2012). “Ecological dynamics and motor learning design in sport” in Skill acquisition in sport: Research, theory and practice. eds. Hodges N. J. Williams A. M. (London: Routledge), 112–130.

[ref22] Davids K. W. Button C. Bennett S. J. (2008). Dynamics of skill acquisition: A constraints-led approach. Champaign, IL: Human Kinetics.

[ref23] de Carvalho E. Rolla G. (2020). An enactive-ecological approach to information and uncertainty. Front. Psychol. 11:588. 10.3389/fpsyg.2020.00588, PMID: 32373006 PMC7186374

[ref24] Delignières D. Fortes M. Ninot G. (2004). The fractal dynamics of self-esteem and physical self. Nonlinear Dynamics Psychol. Life Sci. 8, 479–510. PMID: 15473949

[ref25] Dodel S. Pillai A. Fink P. Muth E. Stripling R. Schmorrow D. (2010). “Observer-independent dynamical measures of team coordination and performance” in Motor control. eds. Danion F. Latash M. L. (Oxford: Oxford University Press), 72–103.

[ref26] Duarte R. Araújo D. Correia V. Davids K. (2012). Sports teams as superorganisms. Sports Med. 42, 633–642. 10.1007/BF03262285, PMID: 22715927

[ref27] Dubois D. (2003). Mathematical foundations of discrete and functional systems with strong and weak anticipations. Lect. Notes Comput. Sci. 2684, 110–132. 10.1007/978-3-540-45002-3_7

[ref28] Duda J. L. Appleton P. R. (2016). “Empowering and disempowering coaching climates: conceptualization, measurement considerations, and intervention implications” in Sport and exercise psychology research. eds. Raab M. Elbe A. -M. Seiler R. Hatzigeorgiadis A. Wylleman P. (London: Academic Press), 373–388.

[ref29] Edelman G. M. Gally J. A. (2001). Degeneracy and complexity in biological systems. Proc. Natl. Acad. Sci. U. S. A. 98, 13763–13768. 10.1073/pnas.231499798 11698650 PMC61115

[ref30] Fajen B. R. Riley M. A. Turvey M. T. (2008). Information, affordances, and the control of action in sport. Int. J. Sport Psychol. 40, 79–107.

[ref31] Fewell J. H. Armbruster D. Ingraham J. Petersen A. Waters J. S. (2012). Basketball teams as strategic networks. PLoS One 7:e47445. 10.1371/journal.pone.0047445, PMID: 23139744 PMC3490980

[ref32] Freytag G. MacEwan E. J. (1908). Freytag’s technique of the drama: An exposition of dramatic composition and art. Chicago: Scott, Foresman and Company.

[ref33] Garcia-Retortillo S. Javierre C. Hristovski R. Ventura J. L. Balagué N. (2019). A principal components analysis as a novel approach for cardiorespiratory exercise testing evaluation. Physiol. Meas. 40:084002. 10.1088/1361-6579/ab2ca0, PMID: 31239421

[ref34] Gibson J. J. Gibson E. J. (1955). Perceptual learning: differentiation or enrichment? Psychol. Rev. 62, 32–41. 10.1037/h0048826, PMID: 14357525

[ref35] Glazier P. S. Davids K. (2009a). The problem of measurement indeterminacy in complex neurobiological movement systems. J. Biomech. 42, 2694–2696. 10.1016/j.jbiomech.2009.08.001 19748625

[ref36] Glazier P. S. Davids K. (2009b). Constraints on the complete optimization of human motion. Sports Med. 39, 15–28. 10.2165/00007256-200939010-00002 19093693

[ref37] Gonçalves B. Coutinho D. Santos S. Lago-Penas C. Jiménez S. Sampaio J. (2017). Exploring team passing networks and player movement dynamics in youth association football. PLoS One 12:e0171156. 10.1371/journal.pone.0171156, PMID: 28141823 PMC5283742

[ref38] Gould D. Diefenbach K. Moffet A. (2002). Psychological characteristics and development in Olympic athletes. J. Appl. Sport Pshychol. 14, 172–204. 10.1371/journal.pone.0171156

[ref39] Haken H. (2006). Information and self-organization: A macroscopic approach to complex systems. New York: Springer Science & Business Media.

[ref40] Hristovski R. (1989). On the dynamics of bio-motor actions as state changes in a human bio-motor field. Fizicka Kultura 43, 59–63.

[ref41] Hristovski R. (2017). “Unpredictability in competitive environments” in Complex systems in sport international congress: Linking theory and practice. eds. Torrents C. Passos P. Cos F. (Barcelona, SA: Frontiers Media), 9–12.

[ref42] Hristovski R. Davids K. Araújo D. (2006). Affordance-controlled bifurcations of action patterns in martial arts. Nonlinear Dynamics Psychol. Life Sci. 10, 409–444. PMID: 16884651

[ref43] Hristovski R. Davids K. Araujo D. (2009). “Information for regulating action in sport: metastability and emergence of tactical solutions under ecological constraints” in Perspectives on cognition and action in sport. eds. Araujo D. Ripoll H. Raab M. (New York: Nova Science Publishers, Inc.), 43–57.

[ref45] Hristovski R. Davids K. Araujo D. Passos P. (2011). Constraints-induced emergence of functional novelty in complex neurobiological systems: a basis for creativity in sport. Nonlinear Dynamics Psychol. Life Sci. 15, 175–206. PMID: 21382260

[ref44] Hristovski R. Davids K. Araújo D. Passos P. Serre N. B. Button C. (eds.) (2013). “Creativity in sport and dance: ecological dynamics on a hierarchically soft-assembled perception–action landscape” in Complex systems in sport (London: Routledge), 287–300.

[ref46] Hristovski R. Dukovski S. (1996). “Morphological latent dimensions interpreted as self-organizing processes of formation of macro-morphological oriented and amorphous dissipative structures” in *Proceedings of the First Scientific Conference on Science and Sport*; October 2-5, 1996; Skopje, Macedonia, 160–164.

[ref47] Hristovski R. Venskaityte E. Vainoras A. Balagué N. Vázquez P. (2010). Constraints controlled metastable dynamics of exercise-induced psychobiological adaptation. Medicina 46, 447–453. 10.3390/medicina46070064, PMID: 20966616

[ref48] Kelso J. S. (2012). Multistability and metastability: understanding dynamic coordination in the brain. Philos. Trans. R. Soc. Lond. Ser. B Biol. Sci. 367, 906–918. 10.1098/rstb.2011.0351, PMID: 22371613 PMC3282307

[ref49] Kiefer A. W. Silva P. L. Harrison H. S. Araújo D. (2018). Antifragility in sport: leveraging adversity to enhance performance. Sport Exerc. Perform. Psychol. 7, 342–350. 10.1037/spy0000130

[ref50] Kreft L. (2012). Sports as a drama. J. Philos. Sport 39, 219–234. 10.1080/00948705.2012.725898

[ref51] Latash M. L. (2008). Synergy. Oxford: Oxford University Press.

[ref88] Latash M. L. Scholz J. P. Schöner G. (2007). Toward a new theory of motor synergies. Motor control 11, 276–308. 10.1123/mcj.11.3.276, PMID: 17715460

[ref52] Layzer D. (1975). The arrow of time. Sci. Am. 233, 56–69. 10.1038/scientificamerican1275-561162324

[ref53] Lennartsson J. Lidström N. Lindberg C. (2015). Game intelligence in team sports. PLoS One 10:e0125453. 10.1371/journal.pone.0125453, PMID: 25970581 PMC4430496

[ref54] Lopes A. M. Tenreiro Machado J. A. (2019). Entropy analysis of soccer dynamics. Entropy 21:187. 10.3390/e21020187 PMC7514670 33266902

[ref55] Lopes A. M. Tenreiro Machado J. A. (2020). Fractional dynamics in soccer leagues. Symmetry 12:356. 10.3390/sym12030356

[ref56] Maldonado G. Bailly F. Souères P. Watier B. (2018). On the coordination of highly dynamic human movements: an extension of the uncontrolled manifold approach applied to precision jump in parkour. Sci. Rep. 8, 1–14. 10.1038/s41598-018-30681-6 30111843 PMC6093881

[ref57] Memmert D. (2015). Teaching tactical creativity in sport: Research and practice. London: Routledge.

[ref58] Memmert D. Baker J. Bertsch C. (2010). Play and practice in the development of sport-specific creativity in team ball sports. High Abil. Stud. 21, 3–18. 10.1080/13598139.2010.488083

[ref59] Mytton G. J. Archer D. T. Turner L. Skorski S. Renfree A. Thompson K. G.. (2015). Increased variability of lap speeds: differentiating medalists and nonmedalists in middle-distance running and swimming events. Int. J. Sports Physiol. Perf. 10, 369–373. 10.1123/ijspp.2014-0207, PMID: 25230099

[ref60] Neuman Y. Israeli N. Vilenchik D. Cohen Y. (2018). The adaptive behavior of a soccer team: an entropy-based analysis. Entropy 20:758. 10.3390/e20100758 PMC7512319 33265847

[ref61] Passos P. Araujo D. Davids K. Gouveia L. Serpa S. Milho J.. (2009). Interpersonal pattern dynamics and adaptive behavior in multi-agent neurobiological systems: a conceptual model and data. J. Mot. Behav. 41, 445–459. 10.3200/35-08-061, PMID: 19482724

[ref62] Passos P. Milho J. Button C. (2018). Quantifying synergies in two-versus-one situations in team sports: an example from Rugby union. Behav. Res. Methods 50, 620–629. 10.3758/s13428-017-0889-3, PMID: 28425057

[ref63] Pol R. Balagué N. Ric A. Torrents C. Hristovski R. Kiely J. (2020). Training or synergizing? Complex systems principles change the understanding of sport processes. Sports Med. Open 6:28. 10.1186/s40798-020-00256-9, PMID: 32661759 PMC7359207

[ref64] Rangel W. Ugrinowitsch C. Lamas L. (2019). Basketball players’ versatility: assessing the diversity of tactical roles. Int. J. Sports Sci. Coach. 14, 552–561. 10.1177/1747954119859683

[ref65] Ric A. Hristovski R. Gonçalves B. Torres L. Sampaio J. Torrents C. (2016). Timescales for exploratorytactical behaviour in football small-sided games. J. Sports Sci. 34, 1723–1730. 10.1080/02640414.2015.1136068, PMID: 26758958

[ref66] Rietveld E. Kiverstein J. (2014). A rich landscape of affordances. Ecol. Psychol. 26, 325–352. 10.1080/10407413.2014.958035

[ref67] Riley M. A. Richardson M. Shockley K. Ramenzoni V. C. (2011). Interpersonal synergies. Front. Psych. 2:38. 10.3389/fpsyg.2011.00038, PMID: 21716606 PMC3110940

[ref68] Rosslee G. J. (2014). Defining and developing a theory of sport intelligence. doctoral dissertation. Pretoria: University of South Africa. Available at: http://hdl.handle.net/10500/18508 (Accessed March 30, 2014).

[ref69] Scholz J. P. Schöner G. (1999). The uncontrolled manifold concept: identifying control variables for a functional task. Exp. Brain Res. 126, 289–306. 10.1007/s002210050738, PMID: 10382616

[ref70] Schöner G. (1995). Recent developments and problems in human movement science and their conceptual implications. Ecol. Psychol. 7, 291–314. 10.1207/s15326969eco0704_5

[ref71] Segundo-Ortin M. Heras-Escribano M. Raja V. (2019). Ecological psychology is radical enough: a reply to radical enactivists. Philos. Psychol. 32, 1001–1023. 10.1080/09515089.2019.1668238

[ref72] Seifert L. Button C. Davids K. (2013). Key properties of expert movement systems in sport: an ecological dynamics perspective. Sports Med. 43, 167–178. 10.1007/s40279-012-0011-z, PMID: 23329604

[ref73] Seifert L. Wattebled L. Herault R. Poizat G. Adé D. Gal-Petitfaux N.. (2014). Neurobiological degeneracy and affordance perception support functional intra-individual variability of inter-limb coordination during ice climbing. PLoS One 9:e89865. 10.1371/journal.pone.0089865, PMID: 24587084 PMC3933688

[ref74] Serdyukov S. I. (1987). “On the change of entropy in models of morphogenesis” in Theoretical and mathematical aspects of morphogenesis. eds. Presnov E. V. Maresin V. M. Zotin A. I. (Moskva: Nauka).

[ref75] Shannon C. E. Weaver W. (1949). The mathematical theory of communication. Illinois: University of Illinois Press.

[ref76] Silva P. Duarte R. Esteves P. Travassos B. Vilar L. (2016). Application of entropy measures to analysis of performance in team sports. Int. J. Perform. Anal. Sport 16, 753–768. 10.1080/24748668.2016.11868921

[ref89] Silva P. Garganta J. Araújo D. Davids K. Aguiar P. (2013). Shared knowledge or shared affordances? Insights from an ecological dynamics approach to team coordination in sports. Sports Med. 43, 765–772. 10.1007/s40279-013-0070-9 23794235

[ref77] Simon H. A. (1956). Rational choice and the structure of the environment. Psychol. Rev. 63, 129–138. 10.1037/h0042769, PMID: 13310708

[ref78] Stepp N. Turvey M. T. (2010). On strong anticipation. Cogn. Syst. Res. 11, 148–164. 10.1016/j.cogsys.2009.03.003, PMID: 20191086 PMC2827858

[ref79] Taleb N. N. Douady R. (2013). Mathematical definition, mapping, and detection of (anti) fragility. Quant. Finance 13, 1677–1689. 10.1080/14697688.2013.800219

[ref80] Thiel C. Foster C. Banzer W. De Koning J. (2012). Pacing in Olympic track races: competitive tactics versus best performance strategy. J. Sports Sci. 30, 1107–1115. 10.1080/02640414.2012.701759, PMID: 22738897

[ref81] Torrents C. Balagué N. Ric Á. Hristovski R. (2020). The motor creativity paradox: constraining to release degrees of freedom. Psychol. Aesthet. Creat. Arts. [Preprint]. 10.1037/aca0000291

[ref82] Turvey M. T. (1992). Affordances and prospective control: an outline of the ontology. Ecol. Psychol. 4, 173–187. 10.1207/s15326969eco0403_3

[ref83] Verkhoshansky Y. Siff M. C. (2009). Supertraining. Rome: Verkhoshansky SSTM.

[ref84] Vilar L. Araújo D. Davids K. Bar-Yam Y. (2013). Science of winning soccer: emergent pattern-forming dynamics in association football. J. Syst. Sci. Complex. 26, 73–84. 10.1007/s11424-013-2286-z

[ref85] Wissner-Gross A. D. Freer C. E. (2013). Causal entropic forces. Phys. Rev. Lett. 110:168702. 10.1103/PhysRevLett.110.168702 23679649

[ref86] Withagen R. (2018). Towards an ecological approach to emotions and the individual differences therein. New Ideas Psychol. 51, 21–26. 10.1016/j.newideapsych.2018.04.004

[ref87] Woolley A. W. Chabris C. F. Pentland A. Hashmi N. Malone T. W. (2010). Evidence for a collective intelligence factor in the performance of human groups. Science 330, 686–688. 10.1126/science.1193147, PMID: 20929725

